# Integrating Microalgal *Chlorella* Biomass and Biorefinery Residues into Sustainable Agriculture and Food Production: Insights from Lettuce Cultivation

**DOI:** 10.3390/foods14050808

**Published:** 2025-02-26

**Authors:** Antira Wichaphian, Apiwit Kamngoen, Wasu Pathom-aree, Wageeporn Maneechote, Tawanchai Khuendee, Yupa Chromkaew, Benjamas Cheirsilp, Douglas J. H. Shyu, Sirasit Srinuanpan

**Affiliations:** 1 Master of Science Program in Applied Microbiology (International Program), Department of Biology, Faculty of Science, Chiang Mai University, Chiang Mai 50200, Thailand; antira_w@cmu.ac.th; 2 Department of Biology, Faculty of Science, Chiang Mai University, Chiang Mai 50200, Thailand; wasu.p@cmu.ac.th (W.P.-a.); wageeporn.m@cmu.ac.th (W.M.); 3 Microbial Biorefinery and Biochemical Process Engineering Research Group, Chiang Mai University, Chiang Mai 50200, Thailand; meanapiwit@gmail.com; 4 Functional Genomics Laboratory, Department of Biological Science and Technology, National Pingtung University of Science and Technology, Neipu, Pingtung 91201, Taiwan; dshyu@mail.npust.edu.tw; 5 Center of Excellence in Microbial Diversity and Sustainable Utilization, Faculty of Science, Chiang Mai University, Chiang Mai 50200, Thailand; yupa.c@cmu.ac.th; 6 Office of Research Administration, Office of the University, Chiang Mai University, Chiang Mai 50200, Thailand; 7 Department of Plant and Soil Sciences, Faculty of Agriculture, Chiang Mai University, Chiang Mai 50200, Thailand; tawanchai_kh@cmu.ac.th; 8 Program of Biotechnology, Faculty of Agro-Industry, Prince of Songkla University, Hat Yai, Songkhla 90110, Thailand; benjamas.che@psu.ac.th

**Keywords:** microalgal biofertilizer, lettuce growth, soil nutrient dynamics, microbial community composition, nitrogen uptake, sustainable agriculture

## Abstract

Microalgal biomass offers a promising biofertilizer option due to its nutrient-rich composition, adaptability, and environmental benefits. This study evaluated the potential of microalgal-based biofertilizers—microalgal *Chlorella* biomass, de-oiled microalgal biomass (DMB), and de-oiled and de-aqueous extract microalgal biomass (DAEMB)—in enhancing lettuce growth, soil nutrient dynamics, and microbial community composition. Lettuce seedlings were cultivated with these biofertilizers, and plant growth parameters, photosynthetic pigments, and nitrogen uptake were assessed. Soil incubation experiments further examined nutrient mineralization rates, while DNA sequencing analyzed shifts in rhizosphere microbial communities. Lettuce grown with these biofertilizers exhibited improved growth parameters compared to controls, with *Chlorella* biomass achieving a 31.89% increase in shoot length, 27.98% in root length, and a 47.33% increase in fresh weight. Chlorophyll a and total chlorophyll levels increased significantly in all treatments, with the highest concentrations observed in the *Chlorella* biomass treatment. Soil mineralization studies revealed that DMB and DAEMB provided a gradual nitrogen release, while *Chlorella* biomass exhibited a rapid nutrient supply. Microbial community analyses revealed shifts in bacterial and fungal diversity, with increased abundance of nitrogen-fixing and nutrient-cycling taxa. Notably, fungal diversity was enriched in biomass and DAEMB treatments, enhancing soil health and reducing pathogenic fungi. These findings highlight microalgal biofertilizers’ potential to enhance soil fertility, plant health, and sustainable resource use in agriculture.

## 1. Introduction

Global agriculture faces a dual challenge of meeting the growing demand for food while minimizing environmental impact. Intensive farming practices have relied heavily on chemical fertilizers to boost crop yields, but this approach has led to severe consequences, including soil degradation, loss of biodiversity, and nutrient runoff into water bodies [1]. These challenges, compounded by a rising global population, necessitate a shift towards sustainable agricultural practices that enhance soil health, reduce reliance on synthetic inputs, and promote environmental sustainability [2].

Among various crops, lettuce (*Lactuca sativa*), a widely cultivated leafy vegetable, is particularly representative of these challenges. Renowned for its high nutritional value and rapid growth cycle [3], lettuce is a staple in both small-scale and commercial agricultural systems. Its cultivation, however, often relies heavily on nitrogen (N)-intensive inputs to achieve optimal yields [4]. This dependency not only exacerbates environmental concerns but also highlights the pressing need for sustainable and economically viable alternatives to conventional farming practices. As such, lettuce serves as an ideal model for evaluating innovative agricultural solutions that balance productivity with ecological stewardship.

Biofertilizers, which include microbial inoculants, organic soil conditioners, and natural plant growth stimulants, have emerged as eco-friendly alternatives to chemical fertilizers [5]. However, their widespread adoption has been hindered by high production costs and inconsistent performance under varying field conditions. This has driven interest in microalgal biofertilizers, particularly microalga *Chlorella*-based biofertilizers, which hold great promise for sustainable agriculture due to their rich nutrient profile, rapid growth, and adaptability to diverse environments [6]. Microalgae are versatile organisms with potential applications extending beyond agriculture to include biofuel production and wastewater treatment. When cultivated using nutrient-rich waste streams, microalgae can simultaneously address waste management challenges and produce valuable biomass [7].

*Chlorella*-based biofertilizers offer distinct advantages over conventional biofertilizers. They provide a well-balanced macronutrient profile, including significant amounts of nitrogen (N), phosphorus (P), and organic matter (OM), with enhanced bioavailability due to their rich composition of proteins, polysaccharides, and micronutrients. These nutrients are gradually released through microbial decomposition, supporting sustained plant growth [8]. *Chlorella* biomass also significantly alters rhizosphere microbial communities by increasing the abundance of nitrogen-fixing and nutrient-cycling bacteria and fungi, such as *Proteobacteria*, *Actinobacteria*, and *Ascomycota*. This microbial stimulation surpasses that of many conventional biofertilizers, leading to improved long-term nutrient availability and soil structure [2,5,8]. Additionally, from a sustainability perspective, *Chlorella*-based biofertilizers contribute to a circular bioeconomy by utilizing residual biomass from biorefinery processes, reducing agricultural waste, and minimizing environmental impact [8]. Unlike traditional organic biofertilizers, which may require extended decomposition periods or pose pathogen risks, *Chlorella*-based biofertilizers offer a cleaner, more sustainable alternative with predictable nutrient release rates, mitigating issues such as nitrate leaching, greenhouse gas emissions, and soil degradation associated with synthetic fertilizers.

Despite these advantages, the agricultural application of microalgae has been limited by the underutilization of residual biomass generated during biorefinery processes. For instance, after lipid extraction for biodiesel production, a significant portion of the biomass—rich in N and P—remains as a byproduct [9]. This de-oiled biomass, if repurposed as a biofertilizer, could improve resource efficiency while reducing waste, aligning with circular bioeconomy principles [10]. However, there is a critical gap in research evaluating the agronomic potential of these residues under real-world cultivation scenarios.

This study addresses these gaps by investigating the integration of microalgal *Chlorella* biomass and biorefinery residues into sustainable agriculture through their application as biofertilizers in lettuce cultivation. Specifically, three types of microalgal biofertilizers—whole *Chlorella* biomass, de-oiled microalgal biomass (DMB), and de-oiled and de-aqueous extract microalgal biomass (DAEMB)—are evaluated for their nutrient release profiles during decomposition and their influence on soil microbial dynamics. By assessing their impacts on plant growth, soil health, and microbial communities, this research highlights their potential as cost-effective and eco-friendly alternatives to chemical fertilizers.

## 2. Materials and Methods

### 2.1. Material Source and Seed Preparation

The biomass of the microalga *Chlorella pyrenoidosa* (total nitrogen = 6.69%, total phosphorus = 4.31%, total potassium = 0.18%, total calcium = 0.52%, total magnesium = 0.34%, organic matter = 72.78%), used in this study as a biofertilizer, was purchased from Earth Circle Organics company (Lot #663701, Las Vegas, NV, USA). The de-oiled microalgal biomass (DMB) (total nitrogen = 8.47%, total phosphorus = 2.34%, total potassium = 0.002%, total calcium = 0.28%, total magnesium = 0.05%, organic matter = 52.73%) was obtained through lipid extraction from biorefinery processes, as described in our previous study [11]. Similarly, the de-oiled and de-aqueous extract microalgal biomass (DAEMB) (total nitrogen = 8.07%, total phosphorus = 1.94%, total potassium = 0.02%, total calcium = 0.42%, total magnesium = 0.17%, organic matter = 58.53%) was prepared by subjecting the biomass to both lipid extraction and aqueous extraction processes, as detailed in the same study [12]. The lettuce seeds were obtained from the Vegetable Seed Production and Organic Farming Learning Center at Maejo University, Thailand. Prior to use, the seeds were sterilized through a disinfection protocol involving immersion in 70% ethanol (*v*/*v*) for one minute, followed by soaking in 1.2% sodium hypochlorite (*v*/*v*) for 12 min, and finally rinsing three times with sterile deionized water. These sterilized seeds were then utilized in the seedling experiment.

### 2.2. Seedling Experiment

The DMB and DAEMB were grounded using a mortar and pestle, and peat moss (Potground H. Klasmann, Klasmann-Deilmann Company, Geeste, Germany) was used as potting soil. The experiments were divided into four treatments: (1) Control: 1 kg of peat moss; (2) Biomass: 1 kg of peat moss mixed with 0.5 g of *Chlorella* biomass; (3) DMB: 1 kg of peat moss mixed with 0.5 g of DMB; (4) DAEMB: 1 kg of peat moss mixed with 0.5 g of DAEMB. This study employed a standardized quantity of 0.5 g/kg soil for the DMB, guided by the optimal amount recommended in the reports of Silambarasan et al. [8]. The sterilized lettuce seeds were sown in a seedling pot. When the first true leaves appeared, the seedlings were moved into seedling trays with each treatment, with one seedling per cell. Each treatment, with a total of 30 seedlings per treatment, was placed in a greenhouse, exposed to natural sunlight (12 h photoperiod, 306–2311 µmol m^−2^ s^−1^ photosynthetically active radiation, 20–27 °C temperature range, and 61–76% relative humidity range), and irrigated with tap water. After 7 days, 1 mL of *Streptomyces thermocarboxydus* S3 spore solution at a concentration of 10^8^ spores per milliliter was added to each cell according to the treatment. The decision to inoculate lettuce seeds with *S. thermocarboxydus* S3 was guided by earlier research findings. Previous studies revealed that this actinomycete effectively mitigates the adverse effects of nutrient stress on lettuce [13]. Additionally, lettuce plants treated with this strain exhibited notable enhancements in growth metrics, such as increased fresh weight, longer root length, and greater overall plant length [13,14]. The lettuce seedlings were harvested after 25 days of planting.

### 2.3. Determination of Plant Growth Parameters

Plant growth parameters, including shoot length, root length, fresh weight, dry weight, and the number of leaves, were evaluated. Chlorophyll a, chlorophyll b, total chlorophyll, and carotenoid content were measured according to the method of Arnon [15].

### 2.4. Determination of Nutrient Uptake

A 0.5 g dried lettuce sample of leaves and/or roots was mixed with an H_2_SO_4_-Na_2_SO_4_-Se solution and digested using a Kjeldatherm (KT 20s, Gerhardt, Germany) at a temperature range of 360–400 °C for 3–4 h. After digestion, the solution was allowed to cool, filtered, and mixed with 2 mL of the extract and 0.5 mL of sodium-EDTA reagent, followed by thorough mixing. Next, 2 mL of salicylate-nitroprusside reagent was added, and the mixture was again thoroughly mixed. One milliliter of buffered hypochlorite reagent was then introduced, followed by thorough mixing. The sample was incubated at room temperature for 2 h, after which the absorbance was measured at 667 nm. The nitrogen concentration was determined by comparing the absorbance values to a standard calibration curve. The total nitrogen content and nitrogen uptake in the samples were calculated using the following Equation (1) [16] and Equation (2) [17], respectively:

Total N (%) = (C × V)/(W × 10^4^) (1)

N uptake = (N_sample_ × DW)/100 (2)

where C is the nitrogen concentration in the sample (mg/kg), determined from the standard curve, V is the total volume of the sample solution from the digestion process (mL), W is the sample weight (g), N_sample_ is the total nitrogen content in the samples (leaves or roots), and DW is the dry weight of the lettuce samples.

### 2.5. Determination of Mineralization

#### 2.5.1. Soil Sampling

The soil samples were collected from the Agricultural Innovation Research, Integration, Demonstration and Training Center. Stones and wood fragments were removed, and the soil was sieved through a 2 mm mesh to eliminate any debris.

#### 2.5.2. Soil Incubation

The soil incubation experiment was conducted at the Plant and Soil Laboratory, Chiang Mai University, at room temperature. The treatments were divided into seven groups: (1) Control: 100 g of dried soil, (2) Biomass: 100 g of dried soil + 1 g of *Chlorella* biomass, (3) DMB: 100 g dried soil + 1 g of DMB, (4) DAEMB: 100 g of dried soil + 1 g of DAEMB, (5) Biomass-S3: 100 g of dried soil + 1 g of *Chlorella* biomass + 1 mL of *S. thermocarboxydus* S3 spores solution, (6) DMB-S3: 100 g of dried soil + DMB + 1 mL of *S. thermocarboxydus* S3 spores solution, and (7) DAEMB-S3: 100 g of dried soil + DAEMB + 1 mL of *S. thermocarboxydus* S3 spores solution. The components were mixed and placed in containers according to the treatment. All containers were covered with perforated aluminum foil. Each treatment included three replications. Soil moisture was maintained at 45–60% by watering every three days. The soil was incubated for 30 days, and inorganic nitrogen (${\mathrm{NH}}_{4}^{+}$-N and ${\mathrm{NO}}_{3}^{\text{-}}$-N) was analyzed on the first sampling date and on days 7, 14, 21, and 30 after treatment [18].

#### 2.5.3. Determination of Inorganic Nitrogen

Inorganic nitrogen for each sample was determined by extracting 10 g of soil sample with 50 mL of 2M KCl solution. The mixture was shaken for 30 min and filtered using Whatman No. 5 filter paper to obtain a clear extract. Following extraction, the quantification of ${\mathrm{NH}}_{4}^{+}$-N and ${\mathrm{NO}}_{3}^{-}$-N was performed using the colorimetric method [19] and the modified Cataldo method [20], respectively. ${\mathrm{NH}}_{4}^{+}$-N was quantified by mixing 2 mL of the soil extract with 0.5 mL of sodium-EDTA reagent and thoroughly mixing. Next, 2 mL of salicylate-nitroprusside reagent was added, and the mixture was again thoroughly mixed. One milliliter of buffered hypochlorite reagent was then introduced, followed by thorough mixing. The sample was incubated at room temperature for 2 h, after which the absorbance was measured at 667 nm. The ammonium nitrogen concentration was determined by comparing the absorbance values to a standard calibration curve. For ${\mathrm{NO}}_{3}^{-}$-N determination, ${\mathrm{NO}}_{3}^{-}$-N was quantified by mixing 1 mL of soil extract with 0.5 mL of TRI solution and swirling to mix thoroughly. After that, it was dried on a hot plate for approximately 5 min at 100–120 °C and the flasks were cooled. Next, 1 mL of conc. $H_{2}{\mathrm{SO}}_{4}$ was added and swirled and allowed to stand for 5 min. We added 5 mL of DI water and swirled to mix, and allowed the solution to cool. We added 5 mL of 40% *w*/*v* of NaOH solution and swirled to mix. After that, we allowed the solution to cool, after which the absorbance was measured at 410 nm. The nitrate nitrogen concentration was determined by comparing the absorbance values to a standard calibration curve. The total inorganic nitrogen concentration was calculated by the sum of ${\mathrm{NH}}_{4}^{+}$-N and ${\mathrm{NO}}_{3}^{-}$-N concentrations. Net nitrogen mineralization was calculated as the difference in total inorganic nitrogen content between the initial and incubated samples [21], as shown in Equation (3):

(3) $$\mathrm{Net}\;N\;\mathrm{mineralization}={\left[([{\mathrm{NH}}_{4}^{+}\text{-}N\right]}_{\mathrm{final}}{+[{\mathrm{NO}}_{3}^{-}\text{-}N]}_{\mathrm{final}})-{\left([{\mathrm{NH}}_{4}^{+}\text{-}N\right]}_{\mathrm{initial}}{+[{\mathrm{NO}}_{3}^{-}\text{-}N]}_{\mathrm{initial}})]/\Delta t$$

where Δt is the time interval over which mineralization is measured (day).

### 2.6. DNA Extraction and Sequencing Analysis

Rhizosphere soil samples were collected after the lettuce plants had grown for 25 days under each treatment. Specifically, the lettuce roots were cut with sterilized scissors and vigorously shaken using tweezers to remove loosely adhering soil. The root-adhering rhizosphere soil was then collected, and the samples were refrigerated at −80 °C for further analysis. DNA was extracted from the samples using the DNeasy PowerSoil Pro Kit (Qiagen, Germany). The bacterial 16S rRNA gene V3-V4 region and the fungal ITS gene were each amplified three times using the oligonucleotide primer pairs 341F and 806R [22] and ITS1F-2R [11], respectively. Following purification with a Qiagen Gel Extraction Kit (Qiagen, Germany), the PCR products were sequenced using a HiSeq2500 PE250 Illumina sequencing system (Illumina Inc., San Diego, CA, USA). The raw reads were filtered and processed with Trimmomatic (v0.33) and DADA2. After quality filtering, the sequencing data were analyzed using QIIME2 (Quantitative Insights into Microbial Ecology Version 2). The raw data files for bacteria and fungi have been submitted to the NCBI (National Center for Biotechnology Information) database, with accession numbers SAMN41178430, SAMN45812807, SAMN45812808, SAMN45812809, SAMN45812810, SAMN41178601, SAMN45813437, SAMN45813438, SAMN45813439, and SAMN45813440, respectively. Operational taxonomic units (OTUs) were clustered using a 97% similarity cut-off using SILVA database for bacteria classification and UNITE database for fungal classification, which provides phylum- and genus-level categorization. Alpha diversity indices, including abundance-based coverage estimator (ACE), Chao1 richness, and the Shannon–Wiener index were calculated using Mothur software, v.1.

### 2.7. Statistical Analysis

All data were analyzed statistically using SPSS (v. 22.0). A significance level of *p* < 0.05 was established, and a one-way analysis of variance (ANOVA) was performed, accompanied by Duncan’s multiple range tests.

## 3. Results and Discussions

### 3.1. Effect of Microalgae-Based Biofertilizers on Growth of Lettuce

**Figure 1:**
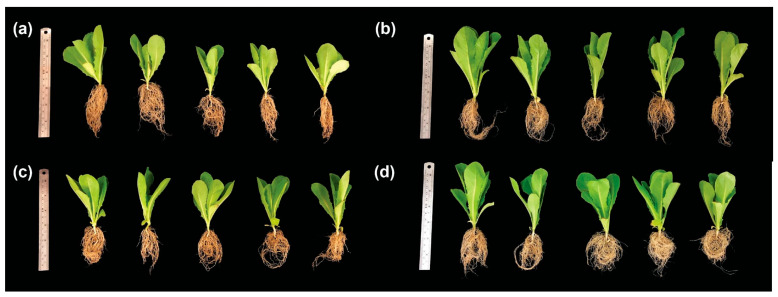
Growth of lettuce from (**a**) soil without microalgal-based biofertilizer, (**b**) soil treated with biomass, (**c**) soil treated with DMB, and (**d**) soil treated with DAEMB.

The effects of various biofertilizer treatments—including soil amended with *Chlorella* sp. biomass (Biomass), soil with de-oiled microalgal biomass (DMB), and soil with de-oiled and de-aqueous extract microalgal biomass (DAEMB)—on lettuce growth, in comparison to soil without any biofertilizer (control), after 25 days of planting are presented in Table 1 and Figure 1. The findings indicate that all three biofertilizer treatments significantly enhanced shoot length, root length, fresh weight, and dry weight compared to the control group, with statistical significance (*p* < 0.05). Among the treatments, the biomass group exhibited the highest growth parameters, with a shoot length of 10.96 cm, a root length of 11.55 cm, a leaf count of 6.60, a fresh weight of 4.23 g, and a dry weight of 0.37 g. These values correspond to increases of 31.89%, 27.91%, 1.54%, 47.39%, and 42.31%, respectively, compared to the control. The DMB treatment also demonstrated high values, with a shoot length of 10.09 cm, a root length of 10.93 cm, a leaf count of 7.40, a fresh weight of 3.81 g, and a dry weight of 0.32 g, reflecting increases of 21.42%, 21.04%, 13.85%, 32.75%, and 23.08%, respectively, relative to the control. Similarly, the DAEMB treatment resulted in a shoot length of 10.23 cm, a root length of 9.46 cm, a leaf count of 6.80, a fresh weight of 3.96 g, and a dry weight of 0.32 g, corresponding to increases of 23.10%, 4.76%, 4.62%, 37.98%, and 23.08%, respectively, compared to the control. The biomass treatment exhibited the highest efficiency in enhancing lettuce growth, likely due to its superior nutrient composition. *Chlorella* biomass contained total nitrogen (6.69%), total phosphorus (4.31%), and organic matter (72.78%), which contributed to its effectiveness. These attributes likely contributed to its superior performance. Nitrogen is crucial for vegetative growth, while phosphorus enhances cell division, root growth, and flowering, playing a key role in energy storage and transfer during photosynthesis [9]. Additionally, the high organic matter content in *Chlorella* biomass may improve soil structure, water retention, and microbial activity, thus creating a more favorable environment for lettuce growth [23].

In comparison, DMB had a higher nitrogen content (8.47%) but lower phosphorus (2.34%) and organic matter (52.73%), while DAEMB contained total nitrogen (8.07%), total phosphorus (1.94%), and organic matter (58.53%), along with additional minerals such as potassium, calcium, and magnesium. Interestingly, the DMB treatment resulted in the highest number of leaves, likely due to its elevated nitrogen content, which plays a crucial role in protein and chlorophyll synthesis—key factors in promoting vigorous leaf development [24]. Moreover, the DAEMB treatment significantly improved plant growth compared to the control, possibly due to its additional nutrients. However, its effectiveness was lower than that of *Chlorella* biomass and DMB, which may be attributed to the de-aqueous extraction process involved in its preparation. This process could have removed essential nutrients and bioactive compounds, thereby reducing its plant growth-promoting effects. In particular, the loss of polysaccharide, amino acid, and phytohormones may have contributed to the slightly lower growth performance observed in DAEMB-treated plants compared to the other treatments [25].

These findings indicate that microalgae biomass can positively enhance lettuce growth, highlighting its potential as an innovative biological fertilizer. Previous research has explored the potential of microalgae as biofertilizers. For instance, Saadaoui et al. [26] demonstrated that soil enrichment with low concentrations (0.25–0.5 g) of algae biomass improved survival rates and shoot length in date palm (*Phoenix dactylifera* L.). Notably, the application of 0.5 g of algae led to the highest root count, the maximum leaf number, and the greatest stem thickness. Similarly, Dineshkumar et al. [27] observed that the incorporation of *Spirulina platensis* and *Chlorella vulgaris* improved the germination rate, growth development, and yield of maize (*Zea mays* L.) during its early stages of development. Likewise, Di Mola et al. [28] reported that seaweed extract (SwE) significantly increased the fresh yield of baby lettuce compared to untreated plants across various nitrogen fertilization rates. Furthermore, Mutale-Joan et al. [29] demonstrated that crude bio-extracts (CBEs) derived from *Aphanothece* sp. and *Chlorella ellipsoidea* significantly improved the root length, root weight, and shoot weight of *Solanum lycopersicum* L.

### 3.2. Effect of Microalgae-Based Biofertilizer on Photosynthesis Pigments

Photosynthetic pigments are essential for capturing light energy and driving photosynthesis. These pigments include total chlorophyll (chlorophyll a and chlorophyll b) and total carotenoids. Chlorophyll, as the primary photosynthetic pigment, absorbs light at specific wavelengths, enabling plants to optimize photosynthesis under varying light conditions. Carotenoids, in addition to their role in light absorption, function as antioxidants that protect plants from photodamage and photoinhibition, thereby maintaining photosynthetic efficiency and overall plant health [30]. The effects of different biofertilizers on photosynthetic pigments, including chlorophyll a, chlorophyll b, total chlorophyll, and carotenoid content in lettuce leaves, are presented in Table 2. The results indicate that contents of chlorophyll a, total chlorophyll, and carotenoid content in lettuce leaves treated with biofertilizers were significantly higher than those in the control group, with statistical significance (*p* < 0.05). Among the treatments, lettuce leaves from the biomass treatment exhibited the highest values, with chlorophyll a at 0.196 mg/g, total chlorophyll at 0.287 mg/g, and carotenoid at 0.086 mg/g. In contrast, chlorophyll b content in lettuce leaves from all biofertilizer treatments did not show a significant difference compared to the control group.

The application of microalgal-based biofertilizers, including biomass, DMB, and DAEMB, significantly enhanced the photosynthetic pigment content in lettuce. Notably, all microalgal-based biofertilizer treatments led to increases in chlorophyll a, total chlorophyll, and carotenoids, with the highest pigment levels observed in plants treated with biomass. In contrast, the control group exhibited the lowest pigment content (Table 2). Improved photosynthetic performance is closely linked to overall plant health, productivity, and physiological function [31]. In our study, enhanced photosynthetic efficiency corresponded with improved plant growth parameters, including increased plant height, root length, leaf count, fresh weight, and dry weight, compared to the control treatment (Table 1).

Previous studies have demonstrated that the application of microalgae can enhance photosynthetic content in plants. For instance, Gharib et al. [32] observed that foliar application of *Arthrospira plantensis*, *C. vulgaris*, and *Nannochloropsis salina* on common bean (*Phaseolus vulgaris* L.) resulted in a significantly higher chlorophyll content index compared to the control group. Similarly, Lopes et al. [33] reported an increase in chlorophyll content in common bean (*Phaseolus vulgaris* L.) following treatment with *Desmodesmus abundans*. Furthermore, Cordeiro et al. [34] demonstrated that foliar application of *Asterarcys quadricellulare* enhanced plant growth and increased bulb size and yield in onion cultivars. Dineshkumar et al. [27] emphasized that microalgal extracts are rich in essential amino acids such as phenylalanine, glutamic acid, and glutamine, which play a critical role in protein synthesis and various plant metabolic processes. These amino acids are crucial for chlorophyll production, energy storage, and the activation of antioxidant enzymes that protect the photosynthetic apparatus, thereby contributing to overall plant health and growth [33].

The enhanced performance observed with the *Chlorella* biomass treatment can be attributed to its elevated nitrogen (6.69%) and adequate phosphorus (4.31%) levels, both of which are crucial macronutrients for plant growth and development. Nitrogen is crucial for plant metabolic processes, significantly influencing growth, yield, and chlorophyll production [35]. Similarly, phosphorus is equally essential, as it supports key metabolic functions, including protein synthesis, cell division, respiration, nutrient transport, and photosynthesis [36]. Furthermore, the increase in lettuce growth and photosynthetic pigment levels following *Chlorella* biomass treatment may be attributed to the presence of L-amino acids in *Chlorella*. These amino acids are efficiently absorbed by plants and can contribute to specific metabolic processes such as chlorophyll synthesis and the stimulation of physiological activities [34].

However, despite the high nitrogen content in the DMB and DAEMB treatments, their lower phosphorus levels may have contributed to reduced photosynthetic pigment concentrations compared to the *Chlorella* biomass treatment. Numerous studies have documented the influence of phosphorus on photosynthesis. For instance, Wang et al. [37] reported that increased phosphorus levels enhanced photosynthesis and resulted in greater leaf number and leaf area in canola (*Brassica napus* L.). Similarly, Shi et al. [38] observed that higher phosphorus concentrations (0.1–1 mM) improved leaf area and relative chlorophyll concentration in peanuts (*Arachis hypogaea* L.), as well as increased plant height and total plant dry weight. These findings highlight the critical role of phosphorus in promoting plant growth and optimizing photosynthetic efficiency.

The absence of significant differences in chlorophyll b content among the treatments could suggest that chlorophyll b synthesis is less sensitive to the variations in nutrient content provided by the different biofertilizers. Chlorophyll b acts as an accessory pigment, capturing light energy and transferring it to chlorophyll a. Its synthesis might be regulated differently compared to chlorophyll a and total chlorophyll [39,40]. However, the accumulation of chlorophylls and carotenoids is affected not only by the plant’s physiological, biochemical, and genetic characteristics but also by environmental factors, including light, temperature, and fertilization [41].

The superior performance of algae biomass treatment in terms of chlorophyll a, total chlorophyll, and carotenoid content indicates that a higher balance of nutrients and organic matter significantly boosts overall plant health and photosynthetic capacity. This aligns with the concept that organic matter improves soil properties and nutrient availability, supporting robust plant growth and pigment synthesis. Previous research has shown that organic matter and balanced nutrient applications enhance plant growth and pigment synthesis by improving soil fertility and structure, increasing microbial activity, and facilitating better nutrient uptake. Studies on the application of nitrogen fertilizers have consistently demonstrated their critical role in chlorophyll synthesis and overall plant health, supporting the findings of increased chlorophyll content in biofertilizer-treated lettuce.

### 3.3. Effect of Microalgal-Based Biofertilizer on Nitrogen Uptake in Lettuce

Nitrogen (N) uptake by lettuce plants was significantly influenced by the application of microalgal-based biofertilizers. As presented in Table 3, the N uptake in lettuce grown in soil without microalgal-based biofertilizer was comparatively lower. The application of microalgal-based biofertilizers positively enhanced N uptake in lettuce. Specifically, the inoculation with biomass and DAEMB resulted in greater N uptake in lettuce leaves compared to the roots. In contrast, inoculation with DMB led to higher N uptake in the roots of lettuce. Furthermore, the application of biomass, DMB, and DAEMB significantly increased N uptake in lettuce leaves by 9.9–16.8%. Notably, the inoculation with DMB enhanced N uptake in lettuce roots by 34.9%, whereas biomass and DAEMB did not exhibit a statistically significant difference in root N uptake compared to the control.

The N uptake of lettuce plants was enhanced by the application of microalgal-based biofertilizers in soil, irrespective of the nitrogen supply, compared to lettuce grown in soil without microalgal-based biofertilizer. A similar increase in N concentration in plant tissues has been reported by Di Mola et al. [42]. The enhanced N uptake, particularly in lettuce leaves, contributed to an increase in photosynthetic pigments. Improved leaf status in terms of nitrate content triggers a more efficient translocation of assimilates to potential photosynthetic sinks, thus boosting plant growth and yield [28,42]. Nitrogen plays a critical role as a primary component of photosynthetic pigments, especially chlorophyll.

Higher nitrogen content in leaves in lettuce treated with biomass, DMB, and DAEMB leads to increased chlorophyll levels, enabling the plant to absorb more light and perform photosynthesis more efficiently, ultimately promoting plant growth [43]. Moreover, nitrogen uptake in the roots was lower in the control and DAEMB treatments, which may explain the reduced root length observed in these groups. This reduced uptake contrasts with the higher nitrogen absorption seen in treatments with biomass and DMB, which likely provided a more consistent nitrogen source. The superior growth parameters observed in the DMB treatment, compared to the control, can be attributed to increased nitrogen uptake in the leaves, facilitated by the additional nitrogen source supplied by DMB. In contrast, the control treatment lacked an external nitrogen source, limiting overall plant growth and development.

### 3.4. Correlation Between Plant Growth and Nitrogen Uptake

**Figure 2:**
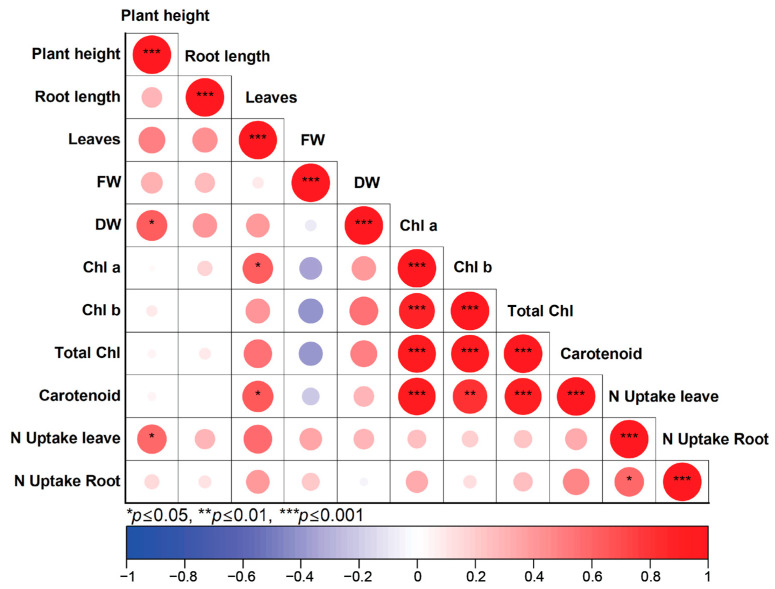
Correlation coefficient between plant growth and nitrogen uptake.

Pearson’s correlation analysis was used to analyze relationships between the measured variables. According to Figure 2, plant height was significantly positively correlated with N uptake in lettuce leaves and the correlation coefficient was 0.59 (*p* < 0.05). The N uptake in lettuce root was significantly positively correlated with the N uptake in lettuce leaves, and the correlation coefficient was 0.58 (*p* < 0.05). Moreover, plant height was significantly positively correlated with dry weight, and the correlation was 0.64 (*p* < 0.05). A significant positive correlation was observed between N uptake in leaves and plant height, highlighting the importance of N uptake in leaves for plant height development. Nitrogen is a critical macronutrient for plants, essential for synthesizing proteins, nucleic acids, and chlorophyll. These compounds are vital for cellular growth, photosynthesis, and metabolic processes [44]. Increased nitrogen uptake enhances plant height by supporting cell division, elongation, and overall plant vigor [42].

As presented in Table 3 and Figure 2, lettuce treated with biofertilizers exhibited higher N uptake and greater plant height compared to lettuce grown in soil without biofertilizer. This outcome is attributed to the higher nitrogen content in soil treated with microalgal-based biofertilizers, providing an accessible nitrogen source for plant uptake and supporting this hypothesis. Additionally, N uptake in leaves was significantly positively correlated with N uptake in roots, indicating that efficient root absorption is a prerequisite for sufficient nitrogen supply to leaves. While root nitrogen uptake positively influences leaf nitrogen uptake, variations in leaf nitrogen content may also arise from factors such as internal nitrogen remobilization, plant-specific nitrogen demand in leaves, and the differential availability and release rates of nitrogen in the soil. These factors contribute to discrepancies in nitrogen uptake between roots and leaves [45]. Furthermore, plant height was positively correlated with dry weight, reflecting the greater structural biomass associated with taller plants. However, no significant relationship was observed between plant height and fresh weight. This is likely because fresh weight measurements are influenced by variable water content in plant tissues, which may not accurately represent the actual biomass [46]. However, fresh weight (FW) exhibited a negative correlation with dry weight (DW), likely due to the influence of water content on FW. DW provides a more accurate reflection of actual biomass accumulation, as a higher FW may primarily indicate increased water retention rather than proportional biomass synthesis. This suggests that plants with greater water content do not necessarily demonstrate enhanced physiological performance in terms of biomass production. Additionally, the observed negative correlation between FW and photosynthetic pigments—including chlorophyll a, chlorophyll b, total chlorophyll, and carotenoids—may be attributed to a dilution effect. In plants with higher water content, the relative concentration of photosynthetic pigments per unit of FW appears lower due to the increased proportion of water within the tissue. This dilution effect can lead to an underestimation of actual pigment levels, despite potentially similar or even greater absolute pigment accumulation [47,48]. A recent study by González-Espíndola and Pedroza-Sandoval [49] further supports this finding, demonstrating that increased tissue water content alters pigment concentration measurements and emphasizes the need to account for dilution effects when assessing photosynthetic capacity.

### 3.5. Soil Mineralization

This study included seven soil treatments: (1) control (no biofertilizer), (2) biomass, (3) de-oiled microalgal biomass (DMB), (4) de-oiled and de-aqueous extract microalgal biomass (DAEMB), (5) biomass-S3 (biomass + *S. thermocarboxydus* S3), (6) DMB-S3 (DMB + *S. thermocarboxydus* S3), and (7) DAEMB-S3 (DAEMB + *S. thermocarboxydus* S3). The inclusion of both non-inoculated and S3-inoculated treatments was aimed at determining whether microalgal-based biofertilizers alone could enhance soil fertility and evaluating the role of *S. thermocarboxydus* S3 in nitrogen mineralization.

#### 3.5.1. Soil N Content

**Figure 3:**
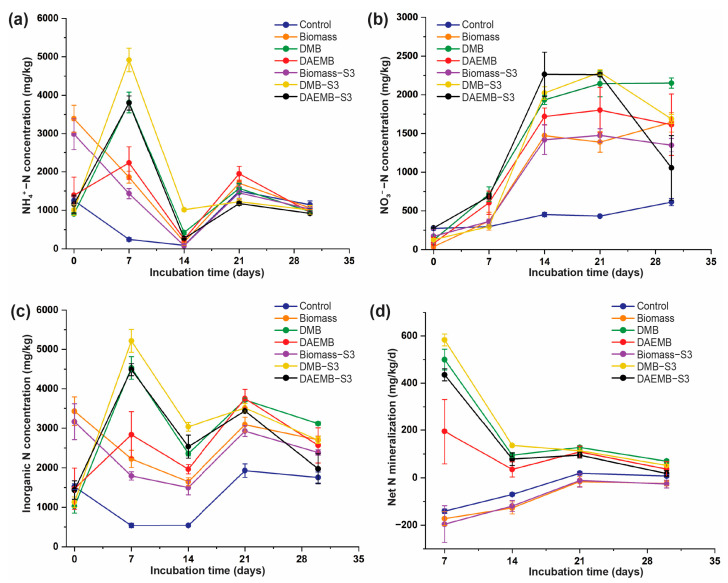
Concentration of ammonium nitrogen (**a**), nitrate nitrogen (**b**), and inorganic nitrogen (**c**), and net nitrogen mineralization (**d**) of soil after 30 days of incubation. The treatments are as follows: Control: Soil without microalgal-based biofertilizer. Biomass: Soil + microalgal *Chlorella* biomass. DMB: Soil + de-oiled microalgal *Chlorella* biomass. DAEMB: Soil + de-oiled and de-aqueous microalgal *Chlorella* biomass. Biomass-S3: Soil + *Chlorella* biomass + *Streptomyces thermocarboxydus* S3. DMB-S3: Soil + DMB + *S. thermocarboxydus* S3. DAEMB-S3: Soil + DAEMB + *S. thermocarboxydus* S3.

The ammonium content across all treatments showed varying patterns, which can be divided into two groups. As shown in Figure 3a, the biomass and biomass-S3 treatments exhibited higher initial ${\mathrm{NH}}_{4}^{+}$-N concentrations (2990.59–3388.47 mg/kg) compared to the control, DMB, DAEMB, DMB-S3, and DAEMB-S3 treatments (903.55–1389.57 mg/kg). During the incubation period, the ${\mathrm{NH}}_{4}^{+}$-N content in the biomass and biomass-S3 treatments decreased to a minimum value on the 14th day (79.29–175.33 mg/kg), followed by an increase to 1451.66–1698.75 mg/kg by the 21st day, and subsequently declined to 1031.96–1061.96 mg/kg by the 30th day, following a trend similar to the control treatment, while the ${\mathrm{NH}}_{4}^{+}$-N content in the DMB, DAEMB, DMB-S3, and DAEMB-S3 treatments exhibited a fluctuating trend. Ammonium levels initially increased from 903.55 to 1389.57 to 2230.13–4917.81 mg/kg by the 7th day, decreased to 245.40–1013.17 mg/kg by the 14th day, rose again to 1175.02–1948.06 mg/kg on the 21st day, and finally decreased to 918.84–1009.23 mg/kg by the end of the incubation period.

In contrast, the ${\mathrm{NO}}_{3}^{-}$-N content showed distinct trends across treatments. In the DMB, DAEMB, and biomass-S3 treatments, ${\mathrm{NO}}_{3}^{-}$-N concentration continuously increased from 81.83 to 173.17 mg/kg to 1419.96 to 1934.03 mg/kg during the initial days up to the 14th day of incubation. Thereafter, in the DMB treatment, the ${\mathrm{NO}}_{3}^{-}$-N level gradually increased to 2151.47 mg/kg by the 30th day. However, in the DAEMB and biomass-S3 treatments, ${\mathrm{NO}}_{3}^{-}$-N content decreased to 1350.10–1613.26 mg/kg, although this change was not significant. For the case of the DMB-S3 and DAEMB-S3 treatments, ${\mathrm{NO}}_{3}^{-}$-N content initially rose from 128.05 to 279.49 mg/kg to 2260.00 to 2289.68 mg/kg, before decreasing to 1054.96 to 1688.51 mg/kg by the end of incubation period. Meanwhile, the biomass treatment exhibited a continuous increase in ${\mathrm{NO}}_{3}^{-}$-N content, rising steadily from 39.45 to 1644.48 mg/kg throughout the incubation period. Similarly, the control treatment showed a gradual increase in ${\mathrm{NO}}_{3}^{-}$-N content from 273.35 to 612.44 mg/kg over the entire incubation period.

The results demonstrated that *S. thermocarboxydus* S3 had minimal effects on nitrogen mineralization in biomass treatments, likely due to the high organic matter (72.78%) and lower nitrogen content (6.69%), which reduced nitrogen bioavailability. In contrast, nitrogen mineralization was significantly enhanced in the DMB and DAEMB treatments when inoculated with *S. thermocarboxydus* S3. ${\mathrm{NH}}_{4}^{+}$-N concentrations were notably higher in DMB-S3 and DAEMB-S3, particularly in the early incubation stages, suggesting that *S. thermocarboxydus* S3 facilitated organic matter decomposition and ammonium release. The subsequent increase in ${\mathrm{NO}}_{3}^{-}$-N at later time points indicated enhanced nitrification, as previously reported for other *Streptomyces* species [50,51].

The high initial ammonium levels of biomass and biomass-S3 treatments likely contained significant amounts of readily mineralizable organic nitrogen, leading to high ammonium concentration at the start. For explanation, the declining amount of ammonium from the initial day to 14th day after incubation may be attributed to its rapid conversion to nitrate by nitrifying bacteria in the soil, such as *Nitrosomonas* and *Nitrobacter.* These microbes grow in aerobic environments and actively oxidize ammonium [52]. Moreover, soil microbes might utilize the available ${\mathrm{NH}}_{4}^{+}$ for their own growth during this phase. This would reduce the pool of bioavailable ammonium in soil [53,54]. The increase in ${\mathrm{NH}}_{4}^{+}$-N concentrations on the 21st day after incubation could result from the decomposition of organic nitrogen compound presence in the microalgal biofertilizer. This process releases ammonium back into the soil as microbial activity breaks down complex nitrogenous materials [55,56]. Moreover, organic nitrogen that was initially more resistant to decomposition might have started breaking down, releasing ${\mathrm{NH}}_{4}^{+}$-N into the soil [55]. Environmental factors might have slowed nitrification, which allowed ${\mathrm{NH}}_{4}^{+}$-N to accumulate. The final declining of ${\mathrm{NH}}_{4}^{+}$-N content, which was found at the end of the incubation periods, could again be driven by the nitrification process. As microbial populations rebound, they may resume converting the newly mineralized ammonium to nitrate. Moreover, ammonium may also re-immobilize into microbial biomass, further reducing its concentration [52,57]. While the DMB, DAEMB, DMB-S3, and DAEMB-S3 showed moderate initial ${\mathrm{NH}}_{4}^{+}$-N concentration, the DMB and DAEMB treatment likely had less readily available organic nitrogen than the biomass treatments, causing more gradual mineralization. The initial increase may indicate that nitrification was not fully active, allowing ${\mathrm{NH}}_{4}^{+}$-N to accumulate [58]. Although there was a decline in ammonium at the 14th day of incubation, ammonium might have been rapidly converted into nitrate, leading to a drop in ${\mathrm{NH}}_{4}^{+}$-N concentrations. Moreover, active microbial uptake of ${\mathrm{NH}}_{4}^{+}$-N for growth could also contribute to its reduction [53]. At the 21st day of incubation, the ${\mathrm{NH}}_{4}^{+}$-N concentration increased; this might have been because the more complex organic compounds in DMB/DAEMB treatment might have begun to break down, releasing additional ${\mathrm{NH}}_{4}^{+}$-N [55]. At the decline of ${\mathrm{NH}}_{4}^{+}$-N at the 30th of incubation, as microbial activity adjusted, nitrification likely resumed, depleting ${\mathrm{NH}}_{4}^{+}$-N concentration. The result showed the difference between biomass treatment and DMB/DAEMB treatment in the case of initial ${\mathrm{NH}}_{4}^{+}$-N levels, fluctuation patterns, and peak and timing; biomass treatment that started with higher concentrations indicated richer nitrogen sources [57], while DMB/DAEMB treatments showed more notable fluctuations, likely due to slower nitrogen release and variable nitrification rates. Furthermore, the timing of ${\mathrm{NH}}_{4}^{+}$-N peaks and declines in DMB/DAEMB suggests a more complex interaction between mineralization and nitrification compared to biomass treatment [55].

The organic matter (OM) content also has an effect on N mineralization. The high OM content led to faster N release; in this case the biomass with 70% OM content provided more organic material for microbial decomposition compared to DMB and DAEMB (50–60% OM content). OM serves as the energy and carbon source for microbes and facilitates rapid mineralization of nitrogen compounds, such as proteins and amino acids [52]. This results in a faster initial release of ammonium during decomposition in biomass treatment. Moreover, the higher OM content serves as a reservoir of organic N which microbes can access over time, causing releasing N at later stages [52,57], contributing to the observed increase in ${\mathrm{NH}}_{4}^{+}$-N around the 21st day in biomass treatment.

Nitrate content is primarily a product of the nitrification process. As shown in Figure 3b, the higher ${\mathrm{NO}}_{3}^{-}$-N content observed on the 14th day of incubation is directly associated with the lower ${\mathrm{NH}}_{4}^{+}$-N content on the same day, indicating that ${\mathrm{NH}}_{4}^{+}$-N was actively converted into ${\mathrm{NO}}_{3}^{-}$-N through nitrification [21]. This trend was particularly evident in biofertilizer treatments including biomass, DMB, DAEMB, biomass-S3, DMB-S3, and DAEMB-S3. By the end of the incubation period, however, the significant decrease in ${\mathrm{NO}}_{3}^{-}$-N content in the DMB-S3 and DAEMB-S3 treatments can be attributed to microbial immobilization, where soil microbes assimilate nitrate for growth as the availability of easily degradable carbon from organic matter declines [53]. Furthermore, as ${\mathrm{NH}}_{4}^{+}$-N levels diminished over time, the nitrification process slowed, resulting in reduced ${\mathrm{NO}}_{3}^{-}$-N production. In contrast, treatments such as biomass and DMB exhibited a continuous increase in ${\mathrm{NO}}_{3}^{-}$-N content. This trend was likely driven by the steady supply of ${\mathrm{NH}}_{4}^{+}$-N through the ongoing mineralization of organic matter, coupled with minimal losses through leaching or denitrification [55]. Interestingly, the decline in ${\mathrm{NO}}_{3}^{-}$-N content in DAEMB and biomass-S3 treatments after the 14th day was not significant, suggesting partial stabilization of nitrate levels, potentially due to a balance between nitrification and nitrate consumption processes [55].

Inorganic N concentration across all treatments showed varying patterns, which can be divided into two groups similarly to an ammonium content trend. As shown in Figure 3c, the biomass and biomass-S3 treatments showed higher initial inorganic concentration, which decreased to lower concentration at the 14th day, followed by an increase to 1451.66–1698.75 mg/kg by the 21st day, and subsequently a decline to 1031.96–1061.96 mg/kg by the 30th day, following a trend similar to the control treatment, while the inorganic content in the DMB, DAEMB, DMB-S3, and DAEMB-S3 treatments exhibited a fluctuating trend. At the initial day, the inorganic content was 1029.38–1437.76 mg/kg, and subsequently increased to the highest at 2835.28–5215.63 mg/kg on the 7th day after incubation, decreased to 1965.11–3036.20 mg/kg by the 14th day, rose again to 3435.02–3712.52 mg/kg on the 21st day, and finally decreased to 1973.80–31140.11 mg/kg by the end of the incubation period. The inorganic concentration trend showed that in DMB and DAEMB treatment, inorganic nitrogen was released slower compared to biomass treatment. Untreated biomass contains a comprehensive profile of organic compounds, including lipids, proteins, and carbohydrates, which are more likely to degrade rapidly due to their intact and native state. In contrast, de-oiled and de-aqueous extracted residues (DMB and DAEMB) undergo processing that significantly alters their chemical composition and structural integrity. The removal of lipids and other extractable components likely reduces the bioavailability of the resulting matrix, leading to prolonged degradation times. Furthermore, ultrasonication and drying processes can cause structural modifications to cell walls, such as cross-linking or hardening, which may increase the resistance of these residues to microbial degradation [59,60]. Additionally, the processes of ultrasonication and solvent extraction may denature proteins, produce fewer biodegradable byproducts, or alter the structure of remaining organic compounds, thereby further impeding microbial decomposition [61].

The mineralization process refers to the biological transformation of organic compounds into simpler inorganic forms. This biological process is influenced by various factors, including temperature, soil properties, microbial community structure, and the chemical composition of biofertilizers [53]. Ammonium and nitrate are the two primary inorganic nitrogen forms that plants can directly absorb and utilize from the rhizosphere. These forms are absorbed by plant roots from the soil and transported to the shoots via the plant’s vascular system. Once transported, nitrogen accumulates in various plant parts, particularly the leaves, which serve as the primary sites for photosynthesis [62]. Nitrogen is a critical component of chlorophyll, the molecule responsible for capturing light energy during photosynthesis [44]. Sufficient nitrogen availability enhances chlorophyll production, resulting in more efficient photosynthesis. Improved photosynthesis leads to higher energy production, which supports plant growth and development, including increased biomass, enhanced root and shoot growth, and overall improved plant health. In this study, microalgal-based biofertilizers, including *Chlorella* biomass, DMB, and DAEMB, were applied in lettuce cultivation. As illustrated in Figure 3c, soils treated with microalgal-based biofertilizers demonstrated higher concentrations of inorganic nitrogen from day 7 to the end of the incubation period. This observation indicates that biofertilizer application enhances inorganic nitrogen content in the soil, facilitating greater nitrogen uptake by plants. Correspondingly, nitrogen uptake values (Table 3) reveal that the application of biofertilizers significantly improved nitrogen uptake efficiency in lettuce, increasing leaf nitrogen uptake by 9.9–16.8%. This improved nitrogen assimilation likely enhanced chlorophyll synthesis and, consequently, photosynthetic efficiency, contributing to greater energy production and plant growth. Enhanced nitrogen uptake and utilization underscore the potential of microalgal-based biofertilizers in improving plant health and productivity. The findings are consistent with prior research highlighting the effectiveness of microalgal biomass as a sustainable alternative to synthetic fertilizers, particularly in enhancing soil fertility and promoting plant growth. For example, Di Mola et al. [42] demonstrated that the application of legume-derived plant hydrolysates significantly improved nitrogen uptake efficiency and nitrogen use efficiency in spinach and lamb’s lettuce compared to untreated plants. Similarly, Egamberdieva et al. [63] found that biochar amendments increased nitrogen uptake in soybean (*Glycine max* L.) and enhanced overall plant growth. Additionally, Mutale-Joan et al. [29] reported that crude extracts from microalgae and cyanobacteria enhanced nutrient concentrations and stimulated tomato growth. However, the extent of these beneficial effects is influenced by several factors, including soil properties, environmental conditions, and the specific composition of the biofertilizers used. These variables underscore the need for tailored approaches to maximize the effectiveness of microalgal-based biofertilizers under different agricultural and environmental conditions.

#### 3.5.2. Soil N Mineralization

The net nitrogen (N) mineralization rates for the biomass and biomass-S3 treatments exhibited negative values throughout the incubation period. These rates increased from −171.99 to −16.15 mg/kg·d and from −195.58 to −11.26 mg/kg·d, respectively, between the initial day and the 21st day of incubation. However, by the 30th day, the rates decreased to −24.05 and −26.06 mg/kg·d, respectively, following a trend similar to that observed in the control treatment. In contrast, the net N mineralization rates for the DMB, DAEMB and DAEMB-S3 treatments demonstrated a serrated pattern. Initially, these rates ranged from 194.84 to 499.99 mg/kg·d but declined to 35.26–94.78 mg/kg·d by the 14th day of incubation. Subsequently, the rates increased to 95.11–127.77 mg/kg·d by the 21st day, before decreasing again to 17.87–69.49 mg/kg·d by the end of the incubation period. The DMB-S3 treatment showed a distinct pattern, with net N mineralization rates continuously decreasing from 583.43 mg/kg·d at the start to 52.20 mg/kg·d by the end of the incubation period.

The net nitrogen mineralization rate reflects the balance between mineralization and immobilization. A negative net N mineralization rate indicates that immobilization exceeds mineralization, meaning that soil microorganisms are consuming more inorganic nitrogen from the soil for their growth than what is being released through mineralization [64]. As shown in Figure 3d, the biomass and biomass-S3 treatments exhibited negative values, suggesting that microbial demand for nitrogen may be higher than the amount being mineralized from the organic matter. This could be due to the limited availability of easily degradable organic matter, causing microorganisms to rely more on the available inorganic nitrogen and leading to net nitrogen immobilization [55]. Similarly to the findings of Toan et al. [65], the researcher investigated the effects of rice straw and nitrogen-fixing *Bacillus subtilis* on nitrogen mineralization; it was observed that the combination of rice straw and *Bacillus subtilis* treatments resulted in greater nitrogen immobilization than mineralization. These findings suggest that immobilization is more pronounced than the mineralization process when rice straw is applied. In contrast, positive net N mineralization rates observed in the DMB, DAEMB, DMB-S3, and DAEMB-S3 treatments indicate that mineralization exceeds immobilization. This could be attributed to higher organic nitrogen content in these biofertilizers, which provides more nitrogen that can be mineralized into ${\mathrm{NH}}_{4}^{+}$-N and ${\mathrm{NO}}_{3}^{-}$-N [57].

Additionally, net nitrogen mineralization analysis revealed that *S. thermocarboxydus* S3 significantly accelerated nitrogen release in DMB and DAEMB treatments, particularly at days 7 and 14, due to their higher nitrogen content (8.47% and 8.07%, respectively) and lower organic matter. In contrast, biomass treatments exhibited net nitrogen immobilization, with minimal impact from *S. thermocarboxydus* S3 inoculation. By days 21 and 30, nitrogen mineralization declined across all treatments, likely due to microbial immobilization and plant uptake [50,51,66], but inorganic nitrogen remained higher in S3-inoculated treatments. These findings underscore the role of *S. thermocarboxydus* S3 in enhancing nitrogen mineralization, particularly in nitrogen-rich and low-organic matter substrates such as DMB and DAEMB, while having limited effects in high-organic matter biomass treatments.

### 3.6. Microbial Communities

#### 3.6.1. Rhizosphere Microbial Diversity and Richness Analysis

In the present study, the soil treated with different sequences of treatments exhibited bacterial operational taxonomic units (OTUs) of 2946, 2179, 2121, 2088, and 2150 for Day 0, control, biomass, DMB, and DAEMB treatments, respectively. The abundance and diversity of bacterial community alpha diversity were evaluated using established biodiversity indices. The ACE and Chao indices were employed to quantify bacterial abundance, while the Shannon and Simpson indices were used to assess bacterial diversity. As shown in Table 4, the ACE, Chao1, Shannon, and Simpson indices exhibited similar patterns, suggesting that microalgal-based biofertilizer may not have a significant effect on the unity of the bacterial community in the soil. Similar results have been reported in previous studies. For example, Tan et al. [67] reported that organic fertilizer did not improve the microbial community but did increase lettuce biomass. Moreover, Zhang et al. [68] also found that inoculant compost of YM1 as a biofertilizer did not significantly improve microbial community but did increase lettuce plant growth. However, in terms of fungal communities, the number of OTUs was 457, 977, 1504, 731, and 1114 for Day 0, control, biomass, DMB, and DAEMB, respectively. The application of microalgal-based biofertilizers, particularly the biomass and DAEMB treatments, increased the number of fungal OTUs in the rhizosphere. Among the treatments, the biomass treatment exhibited the highest ACE and Chao index values, while DMB had the lowest values compared to DAEMB and the control. A similar trend was observed for the Shannon and Simpson indices, with the biomass treatment showing the highest value and DMB the lowest. These findings indicate that the fungi community may have a strong impact on the growth of lettuce. The fungal community structure changed as a result of the nutrients in the microalgal-based biofertilizer. Several studies have reported that rhizosphere fungal activities significantly influence plant growth, nutrient assimilation, and soil health [69]. The plant growth-promoting effects of rhizosphere fungi have been demonstrated in tomatoes, strawberries, and lettuce [68,70,71]. Additionally, rhizosphere fungi establish a connection between soil and plant roots [72]. Rhizosphere fungi are typically involved in the decomposition of soil organic matter and the release of nutrients, thereby promoting crop growth [73]. In this context, the microalgal-based biofertilizer might influence the fungal rhizosphere through nutrient availability and release rates. The higher abundance and diversity of the fungal community observed in biomass and DAEMB treatments indicate that these treatments contain higher nutrient content and release nutrients more rapidly, enhancing fungal abundance and activity to promote rapid growth [68]. Conversely, the lower abundance and diversity of the fungal community observed in the DMB treatment might be due to its slower nutrient release, which still affects fungal communities but at a slower pace. Additionally, since DMB is obtained after the lipid extraction process, it might retain some chemical residues that could impact the fungal community [74].

The findings indicate that applying microalgal-based biofertilizers influenced the beta diversity of the microbial community. This effect is likely due to the role of nutrient availability in shaping microbial community dynamics. Microalgal-based biofertilizers have a complex and variable nutrient composition. Both bacteria and fungi, essential for nutrient cycling and organic matter decomposition, respond to nutrient concentrations in distinct ways [75]. High nutrient levels tend to increase negative interactions among species, whereas low nutrient levels promote closer associations [76]. Similar observations have been made in previous studies. For example, Yang et al. [5] found that biofertilizers combining *Chlorella vulgaris* and *Bacillus* sp. altered microbial community distribution compared to controls. Similarly, Lv et al. [77] reported that biofertilizers made from *Anabaena circinalis* and *Scenedesmus quadricauda* significantly affected bacterial and fungal communities.

#### 3.6.2. Microbial Community Composition

**Figure 4:**
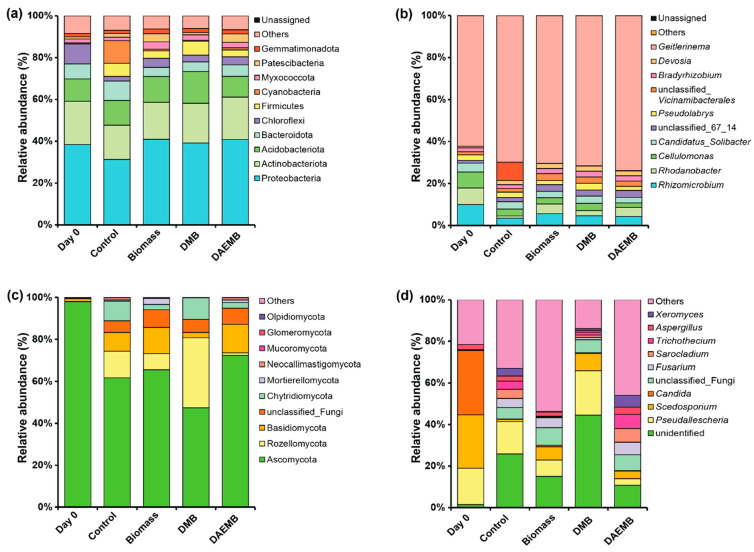
Composition and relative abundance at the phylum and genus level of bacteria (**a**,**b**) and fungi (**c**,**d**).

Microbial communities play a crucial role in decomposing organic matter and mineralizing nutrients, making them more accessible to plants. The dominant bacteria phyla identified in the soil samples were *Proteobacteria* (31.32–40.92%) and *Actinobacteriota* (16.37–20.65%). *Acidiobacteriota* (10.01–15.17%), *Bacteroidota* (4.32–9.27%), and *Chloroflexi* (2.17–9.57%) ranked as the third and fourth most prevalent phyla, respectively (Figure 4a). The findings revealed a higher relative abundance of bacteria from the *Proteobacteria*, *Actinobacteriota*, and *Chloroflexi* groups in soil treated with biomass, DMB, and DAEMB as compared to untreated control soil. The dominant bacterial genera in the sampled soils were *Rhizomicrobium* (3.48–5.57%), *Rhodanobacter* (1.08–4.68%), *Cellulomonas* (2.16–3.41%), and *Candidatus*_*Solibacter* (2.64–3.54%). In soil treated with microalgal biofertilizers, the relative abundance of the *Rhizomicrobium* was notably higher in the biomass treatment (5.57%), DMB treatment (4.62%), and DAEMB treatment (4.26%) compared to the control soil (3.48%). A similar trend was observed for *Rhodanobacter*, with relative abundances of 4.68% in the biomass treatment, 2.48% in the DMB treatment, and 4.33% in the DAEMB treatment, compared to 1.08% in the control soil (Figure 4b).

In this study, the application of microalgal-based biofertilizer, including biomass, DMB, and DAEMB, significantly increased the abundance of *Proteobacteria*, *Actinobacteriota*, *Chloroflexi*, *Rhizomicrobium*, and *Rhodanobacter*. Previous research has reported that *Proteobacteria* are known for their role in nitrogen cycling, particularly in ammonification and nitrification. The enrichment of ammonia-oxidizing bacteria within this group suggests an improved conversion of ammonium to nitrate, enhancing nitrogen availability for plant uptake [78,79,80]. Similarly, *Actinobacteria* contributes to the decomposition of complex organic matter, breaking down proteins and polysaccharides into simpler, plant-available nutrients [5,66,81]. *Chloroflexi* participates in carbon and nitrogen transformations, supporting nutrient mobilization in the rhizosphere [5]. The increased abundance of *Proteobacteria* and *Actinobacteria* compared to the control treatment may be attributed to the addition of the microalgal-based biofertilizer which contains a high amount of nitrogen and organic matter. Concurrently, the observed rise in *Chloroflexi* abundance could be linked to the higher organic matter content [82]. Moreover, mineralization results indicated significant changes in net nitrification within the microalgae-based biofertilizer treatment compared to the control, potentially due to the higher abundance of these microorganisms [80]. *Rhizomicrobium*, a nitrogen-fixing bacterial genus, likely serves as a primary degrader of xylan, facilitating the breakdown of this complex polysaccharide. Other members of the microbial community may benefit from the resultant xylo-oligosaccharides, xylose, or low molecular weight organic compounds derived from this process [83]. The higher abundance of *Rhizomicrobium* in the microalgae-based biofertilizer treatment can be attributed to the rigid cell wall composition of *Chlorella* biomass, which is rich in structural polysaccharides [84]. Also, the increase in *Rhizomicrobium* suggests enhanced nitrogen assimilation in biofertilizer-treated soils. This likely contributed to the higher nitrogen uptake observed in lettuce plants, improving biomass accumulation and chlorophyll content. Moreover, *Rhodanobacter* plays an essential role in the environmental nitrogen cycle, exhibiting complete denitrification along with key plant growth-promoting traits such as the production of indole-3-acetic acid (IAA), siderophores, ammonia, and phosphate solubilization [85,86]. It is also involved in nitrate respiration within the soil nitrogen cycle [87]. In this study, the higher relative abundance of *Rhodanobacter* observed in the microalgae-based biofertilizer treatment may be attributable to the significantly increased nitrogen content of the biofertilizer [87]. Additionally, the presence of *Rhodanobacter*, a bacterial genus known for its denitrification and biocontrol properties, suggests that soil microbiomes were shifting toward a more disease-suppressive state [85,86].

This supports the notion that microalgae-based fertilizers can not only provide essential nutrients but also stimulate microbial populations involved in key biogeochemical processes. The observed increase in microbial diversity, particularly the abundance of nitrogen-fixing and denitrifying bacteria, suggests that microalgae-based biofertilizers could improve soil fertility and enhance plant growth. These changes may promote more sustainable agricultural practices by reducing reliance on synthetic fertilizers. Furthermore, the presence of *Rhodanobacter* with plant growth-promoting traits implies additional benefits for crop productivity, especially in nitrogen-deficient soils. While the study demonstrates a clear link between microalgae-based biofertilizers and microbial community changes, the specific mechanisms driving these shifts remain unclear. Future research should explore the direct interactions between microalgae-derived compounds and soil microbiota. Additionally, long-term field trials are necessary to evaluate the persistence of these microbial changes and their impact on crop yields under varying environmental conditions.

The interactions between microbial community composition and nitrogen mineralization rates in this study reveal how microbial-driven processes influence nitrogen cycling in soils treated with microalgal-based biofertilizers (Biomass, DMB, and DAEMB). Shifts in bacterial and fungal communities were observed in response to these biofertilizers, directly affecting nitrogen mineralization by influencing key processes such as ammonification, nitrification, and denitrification. Among the bacterial contributions, an increased abundance of *Proteobacteria* and *Actinobacteriota* in biofertilizer-treated soils suggests enhanced nitrogen cycling. *Proteobacteria*, particularly ammonia-oxidizing bacteria, facilitate nitrification, converting ammonium into nitrate, while *Actinobacteriota* contribute to the breakdown of organic matter, releasing ammonium through ammonification. Additionally, the presence of *Rhodanobacter*, a known denitrifier, points to active nitrate reduction, especially in biomass and DAEMB treatments. The increased abundance of *Rhizomicrobium*, a nitrogen-fixing genus, further indicates improved nitrogen availability from organic sources in the biofertilizer-treated soils.

The fungal community was predominantly composed of three phyla: *Ascomycota* (47.45–72.37%), *Rozellomycota* (1.17–33.28%), and *Basidiomycota* (2.54–13.65%) (Figure 4c). An increase in the relative abundance of *Ascomycota* and *Basidiomycota* was observed in soils treated with biomass and DAEMB compared to control. Conversely, *Rozellomycota* exhibited a higher relative abundance for the DMB-treated soil compared to the control. Among the identified fungal genera, *Pseudallescheria* (3.18–21.38%) and *Scedosporium* (3.68–8.29%) were the most abundant in soils treated with microalgal biofertilizers. A relative increase in *Pseudallescheria* was observed in the DMB treatment, while *Scedosporium* showed higher relative abundance in the biomass, DMB, and DAEMB treatment compared to control. Additionally, the relative abundance of *Fusarium* increased in biomass (4.76%) and DAEMB (6.06%) treatments but decreased in DMB treatment (1.10%) compared to control (4.39%) (Figure 4d).

Within the fungal community, *Ascomycota* emerged as the dominant phylum across all treatments and constitutes a key component of the lettuce rhizosphere microbiome. This phylum plays a pivotal role in organic matter degradation, soil stabilization, plant biomass decomposition, and endophytic interactions with plants [88]. In this study, the increased relative abundance of *Ascomycota* in biomass and DAEMB treatments may be attributed to the enhanced organic matter content. However, the DMB treatment showed a decrease in the relative abundance of *Ascomycota*, which could indicate a reduction in potential plant pathogenic fungi, such as those in the *Fusarium* genus. This reduction may explain the observed decrease in *Fusarium* abundance in the DMB treatment [89]. Similarly, *Basidiomycota* members are essential for the degradation of lignocellulosic agricultural waste, thereby contributing to carbon and nutrient cycling within soil ecosystems [89]. The increased relative abundances of *Basidiomycota* in biomass and DAEMB treatment can be attributed to organic matter, which may create suitable conditions for the growth of *Basidiomycota*. Both *Ascomycota* and *Basidiomycota* fungi produce extracellular polysaccharides and fungal hyphae, which aid in binding soil particles together, thereby enhancing soil aggregation and porosity. This improved soil structure promotes better aeration, root penetration, and water retention, ultimately reducing drought stress and increasing crop resilience. Additionally, these fungi play a crucial role in lignocellulosic material decomposition, contributing to organic matter stabilization and ensuring long-term soil health and fertility [88,89].

On the other hand, DMB treatment showed significant increasing relative abundance of *Rozellomycota* and *Chytridiomycota*, reporting these microorganisms’ response to organic matter degradation, correlating with higher organic matter levels, as playing an important role in improving soil properties [90,91]. The lower relative abundance of *Ascomycota* and *Basidiomycota* might not necessarily indicate a negative impact on soil health. Instead, the significant increase in the relative abundance of *Rozellomycota* and *Chytridiomycota* could offset this effect, as these fungi are reported to play an important role in improving soil properties [91]. Additionally, the presence of *Rozellomycota* and *Chytridiomycota* suggests that DMB fosters a unique fungal community structure, which may enhance the functional diversity of soil fungi. This, in turn, implies that DMB-treated soil could exhibit higher fungal diversity, contributing to improved soil quality and ecosystem functioning. Among biocontrol fungi, *Pseudallescheria* is a filamentous fungus known to be a potential pathogen for humans and animals, as well as a common soil inhabitant [92], while *Scedosporium* species play a significant role as saprotrophic organisms, contributing to organic matter decomposition. These fungi can serve as indicators of soils with elevated organic matter and nitrogen levels, as their growth is strongly influenced by these conditions [93]. In this study, the increasing relative abundance of *Scedosporium* in biomass, DMB, and DAEMB treatment may be higher for nitrogen and organic matter content in soil compared to the control group. Additionally, the increase in *Rozellomycota* and *Scedosporium* could further enhance biocontrol activity by outcompeting pathogens for nutrients and space in the rhizosphere [90,93]. Lastly, *Fusarium*, particularly the pathogen responsible for Fusarium wilt, has a notable impact on soil ecosystems. Residues from *Fusarium*-related crop diseases increase pathogen populations and accelerate the decomposition of plant residues, particularly diseased ones. This activity is hypothesized to influence soil nutrient cycling by mediating organic matter breakdown and altering soil chemical properties [94].

The results of this study indicate that microalgal-based biofertilizers, such as biomass, DMB, and DAEMB, can significantly alter the fungal community composition, which has important implications for soil health. The increased relative abundance of *Ascomycota* in biomass and DAEMB treatments suggests that these biofertilizers enhance organic matter content, which plays a key role in soil stabilization, nutrient cycling, and plant–microbe interactions. This could contribute to improved soil fertility and ecosystem functioning, providing long-term benefits for agricultural productivity. Moreover, the decline in *Fusarium* abundance in DMB-treated soil suggests that microbial competition or antagonistic interactions may be limiting its proliferation. The higher abundance of beneficial fungi such as *Rozellomycota* and *Chytridiomycota* in the DMB treatment further indicates that DMB fosters a unique fungal community that may enhance soil quality. These fungi are known to respond to organic matter degradation, suggesting that DMB may improve soil properties by promoting the breakdown of organic compounds, enhancing soil structure, and increasing microbial diversity. Additionally, the increased relative abundance of *Basidiomycota* in biomass and DAEMB treatments reflects the potential of these biofertilizers to support fungi involved in the decomposition of lignocellulosic materials, which is essential for carbon and nutrient cycling. Additionally, the presence of beneficial fungi such as *Basidiomycota* further supports root colonization by symbiotic microbes, improving phosphorus solubilization and water absorption [88,89]. The overall shift in fungal community structure indicates that the application of microalgal-based biofertilizers can enhance functional diversity within the soil microbiome, ultimately contributing to a more resilient and sustainable soil ecosystem.

**Figure 5:**
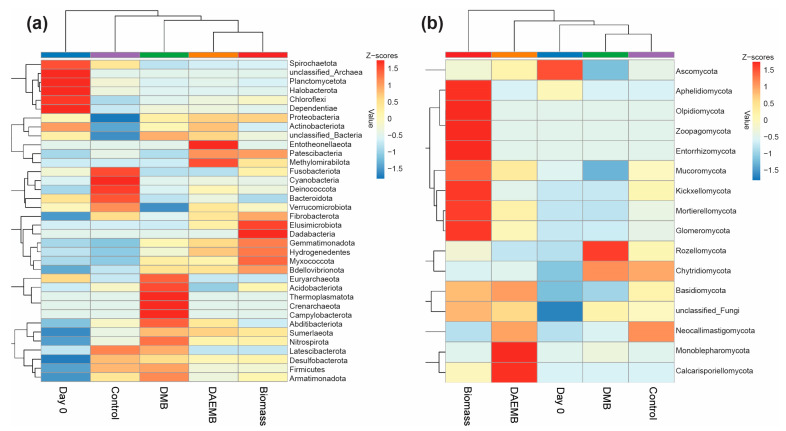
Heatmap analysis of bacterial (**a**) and fungal (**b**) community in lettuce soil treated with different microalgal-based biofertilizers. The color gradient (ranging from blue to red) represents the relative abundance of species, with blue indicating low abundance and red indicating high abundance.

To achieve a more comprehensive understanding of the rhizosphere microbial community associated with lettuce, the community composition was analyzed at the phylum level. As depicted in Figure 5a, the microbial community composition varied significantly across treatments, highlighting distinct impacts of each microalgal-based biofertilizer. In the control treatment, *Cyanobacteria* and *Bacteroidota* were the most abundant phyla, but their relative abundances declined in both the biomass and DMB treatments. The biomass treatment was characterized by a higher relative abundance of *Dadabacteria*, while the DMB treatment showed increased levels of *Thermoplasmatota*, *Crenarchaeota*, and *Campylobacterota*. The DAEMB treatment exhibited a notably higher relative abundance of *Entotheonellaeota* compared to the other treatments. These bacterial taxa are known to contribute to essential soil processes, such as carbon fixation, methane metabolism, and nitrogen and sulfur cycling, which are crucial for maintaining soil health and promoting plant growth [95,96,97,98]. Fungal community composition also varies among treatments (Figure 5b), revealing further insights into the functional roles of these microorganisms. The biomass treatment displayed higher relative abundances of *Olipidiomycota*, *Zoopagomycota*, and *Entorrhizomycota*. *Olipidiomycota* and *Zoopagomycota* are associated with enhanced soil enzyme activity, which supports nutrient mineralization and plant growth [68]. However, *Entorrhizomycota*, while potentially linked to soil nutrient cycling, are also known to cause root swelling disease in cruciferous crops [45], suggesting the need for careful management of their abundance. In the DMB treatment, *Rozellomycota* was particularly abundant, playing an important role in improving soil properties [91]. The DAEMB treatment, in contrast, exhibited elevated levels of *Monoblepharomycota* and *Calcarisporiellomycota*, which are positively correlated with carbon cycling, further enhancing soil fertility [99].

Fungal communities also play a significant role in nitrogen cycling. This study found an increased relative abundance of *Ascomycota* and *Basidiomycota* in biomass and DAEMB treatments, which are both involved in organic matter decomposition and nitrogen release. These fungi are crucial for lignocellulose degradation, breaking down complex organic nitrogen compounds into ammonium. Additionally, *Rozellomycota*, enriched in DMB-treated soils, is linked to organic matter degradation and nitrogen mineralization, which may explain the steady nitrogen release observed in these treatments.

The correlation between microbial shifts and nitrogen mineralization rates showed distinct trends depending on the biofertilizer type, reflecting the influence of specific microbial groups. In biomass treatments, negative net nitrogen mineralization rates were observed early in the incubation period, indicating high microbial nitrogen immobilization. The increased abundance of *Rhodanobacter* in the biomass treatment aligns with active nitrogen immobilization and denitrification, with delayed ammonium peaks suggesting initial microbial nitrogen assimilation followed by organic nitrogen breakdown. Conversely, DMB and DAEMB treatments exhibited fluctuating nitrogen mineralization rates, suggesting a balance between microbial mineralization and immobilization. DMB-treated soils showed higher *Actinobacteria* abundance, which is linked to enhanced ammonification and a more gradual release of inorganic nitrogen, while the increased *Basidiomycota* in DAEMB-treated soils likely contributed to a slow but steady nitrogen mineralization process. These microbial dynamics demonstrate that DMB-treated soil provides a more stable nitrogen source over time, reducing nitrogen losses and ensuring prolonged nutrient availability, which has important agronomic implications.

The observed microbial shifts underscore the potential of microalgal-based biofertilizers to tailor soil microbial communities in ways that improve soil health and plant growth. The dominance of specific bacterial and fungal taxa in each treatment suggests functional specialization, with impacts on carbon and nitrogen cycling, methane metabolism, and enzymatic activity. These processes not only enhance nutrient availability but also contribute to sustainable soil management. By promoting a balanced and diverse microbial ecosystem, *Chlorella*-based biofertilizers further contribute to long-term soil fertility by increasing microbial diversity, which strengthens resilience against environmental stresses like drought, nutrient depletion, and pathogen invasion. Additionally, their ability to sustain nitrogen mineralization over time reduces dependence on synthetic fertilizers, lowering production costs and minimizing environmental impact. Moreover, the unique effects of each biofertilizer highlight the value of integrating microalgal biomass and residues into biorefinery-based agricultural systems, promoting resource efficiency and zero waste.

### 3.7. Limitations and Future Prospects

This study employed a standardized biofertilizer application rate of 0.5 g/kg soil, based on previously reported optimal doses for similar microalgal-based biofertilizers. However, a more comprehensive dose–response analysis would further refine the optimal application rate for each biofertilizer formulation. This study aimed to establish an initial baseline for evaluating the comparative effectiveness of biomass, DMB, and DAEMB. Future research should consider conducting trials with varying biofertilizer concentrations to determine optimal dosages under different soil types and crop conditions. Additionally, further studies could investigate the nutrient release kinetics, shifts in microbial community composition, and long-term impacts on soil health. Understanding these factors will be crucial in optimizing the use of microalgal-based biofertilizers, enhancing their agronomic benefits, and promoting their large-scale adoption in sustainable agriculture. Future research should also integrate chemical fertilizer treatments to evaluate key parameters such as plant growth response, soil fertility dynamics, nutrient release efficiency, and long-term environmental impacts. This approach would provide a clearer perspective on the trade-offs and benefits of microalgal-based biofertilizers in conventional and regenerative agricultural systems, ultimately guiding their optimized use for sustainable crop production. Moreover, the barcode sequencing experiment was conducted without biological replicates, which may limit the generalizability and robustness of the observed microbial community dynamics. Future studies should include multiple replicates to provide more comprehensive and statistically reliable insights. Additional efforts to optimize sequencing depth and coverage should also be considered to minimize potential biases and improve data interpretation.

The cost-effectiveness of microalgae-based fertilizers compared to traditional fertilizers is a crucial consideration for their broader adoption. The price advantage of microalgae-based fertilizers depends on several factors, including the scale of production, the source of raw materials, and the processing technologies used. Currently, microalgae-based fertilizers may have a higher production cost than traditional chemical fertilizers due to the cultivation and harvesting processes. However, advancements in biorefinery techniques, improvements in large-scale cultivation, and the utilization of waste resources for microalgae growth have the potential to significantly reduce these costs over time. Additionally, the long-term benefits of microalgae-based fertilizers, such as their contribution to sustainable agriculture, reduced environmental impact, and improved soil health, may offset the initial higher price.

## 4. Conclusions

This research underscores the efficacy of microalgal-based biofertilizers, particularly *Chlorella* biomass, in enhancing lettuce growth and soil health. The superior nutrient profile and organic matter content of the biofertilizers improved plant development, photosynthetic efficiency, and nitrogen assimilation. Additionally, biofertilizers influenced rhizosphere microbial communities, supporting beneficial microorganisms involved in nutrient cycling and soil stabilization. These outcomes demonstrate the feasibility of repurposing microalgal residues from biofuel production, aligning with sustainable and zero-waste agricultural practices. Therefore, the use of microalgal-based biofertilizers in this study presents a promising strategy to mitigate soil degradation, enhance crop productivity, and promote environmentally sustainable farming. However, future research should focus on optimizing biofertilizer formulations for various crops and conducting long-term field trials to evaluate their ecological and economic impacts.

## Figures and Tables

**Table 1:** Growth parameters of lettuce treated with different microalgal-based biofertilizers.

Treatments	Plant Height (cm)	Root Length (cm)	Number of Leaves	Fresh Weight (g)	Dry Weight (g)
Control	8.31 ± 0.51 ^c^	9.03 ± 0.70 ^c^	6.50 ± 0.76 ^b^	2.87 ± 0.58 ^b^	0.26 ± 0.06 ^b^
Biomass	10.96 ± 0.72 ^a^	11.55 ± 2.40 ^a^	6.60 ± 0.52 ^b^	4.23 ± 0.88 ^a^	0.37 ± 0.10 ^a^
DMB	10.09 ± 0.82 ^b^	10.93 ± 1.64 ^ab^	7.40 ± 0.52 ^a^	3.81 ± 1.03 ^a^	0.32 ± 0.08 ^ab^
DAEMB	10.23 ± 0.43 ^b^	9.46 ± 1.36 ^bc^	6.80 ± 0.63 ^b^	3.96 ± 0.49 ^a^	0.32 ± 0.05 ^ab^

The values in each column are indicated by letters (^a^, ^b^, or ^c^) that are significantly (*p* < 0.05) different from each other based on Duncan’s new multiple range test.

**Table 2:** Photosynthetic pigments of lettuce treated with different microalgal-based biofertilizer.

Treatment	Chlorophyll a (mg/g)	Chlorophyll b (mg/g)	Total Chlorophyll (mg/g)	Carotenoid (mg/g)
Control	0.124 ± 0.028 ^c^	0.069 ± 0.022 ^a^	0.193 ± 0.049 ^b^	0.055 ± 0.011 ^c^
Biomass	0.196 ± 0.034 ^a^	0.091 ± 0.010 ^a^	0.287 ± 0.043 ^a^	0.086 ± 0.011 ^a^
DMB	0.180 ± 0.030 ^ab^	0.102 ± 0.024 ^a^	0.282 ± 0.053 ^a^	0.083 ± 0.013 ^ab^
DAEMB	0.132 ± 0.021 ^bc^	0.069 ± 0.012 ^a^	0.201 ± 0.033 ^ab^	0.064 ± 0.010 ^bc^

The values in each column are indicated by letters (^a^, ^b^, or ^c^) that are significantly (*p* < 0.05) different from each other based on Duncan’s new multiple range test.

**Table 3:** Nitrogen uptake of lettuce treated with microalgal-based biofertilizer.

Treatment	N Leave Uptake (g N/plant)	N Root Uptake (g N/plant)
Control	0.0131 ± 0.0002 ^b^	0.0123 ± 0.0018 ^b^
Biomass	0.0149 ± 0.0009 ^a^	0.0138 ± 0.0005 ^b^
DMB	0.0153 ± 0.0013 ^a^	0.0166 ± 0.0017 ^a^
DAEMB	0.0144 ± 0.0006 ^ab^	0.0119 ± 0.0002 ^b^

The lowercase letters ‘^a^’ and ‘^b^’ denote significant differences (*p* < 0.05) between various samples within each treatment.

**Table 4:** Microbial richness and diversity indices in the rhizosphere soil.

Microbe	Samples	OTUs	ACE	Chao1	Shannon	Simpson
Bacteria	Day 0	2946	3278	3116	8.03	0.9801
	Control	2179	3742	2908	8.68	0.9904
	Biomass	2121	4309	3390	8.93	0.9935
	DMB	2088	3475	2929	9.06	0.9949
	DAEMB	2150	3264	2773	8.95	0.9941
Fungi	Day 0	457	579	567	3.65	0.8627
	Control	977	1079	1077	6.49	0.9602
	Biomass	1504	1707	1729	7.89	0.9867
	DMB	731	962	939	4.25	0.8437
	DAEMB	1114	1188	1223	7.30	0.9823

## Data Availability

The original contributions presented in the study are included in the article, further inquiries can be directed to the corresponding author.
